# Supplementary figures and images for: SARS-CoV-2 variants reveal features critical for replication in primary human cells
Source: PLoS Biol. 2021 Mar 24;19(3):e3001006. doi: 10.1371/journal.pbio.3001006 (PMC8021179; doi:10.1371/journal.pbio.3001006)

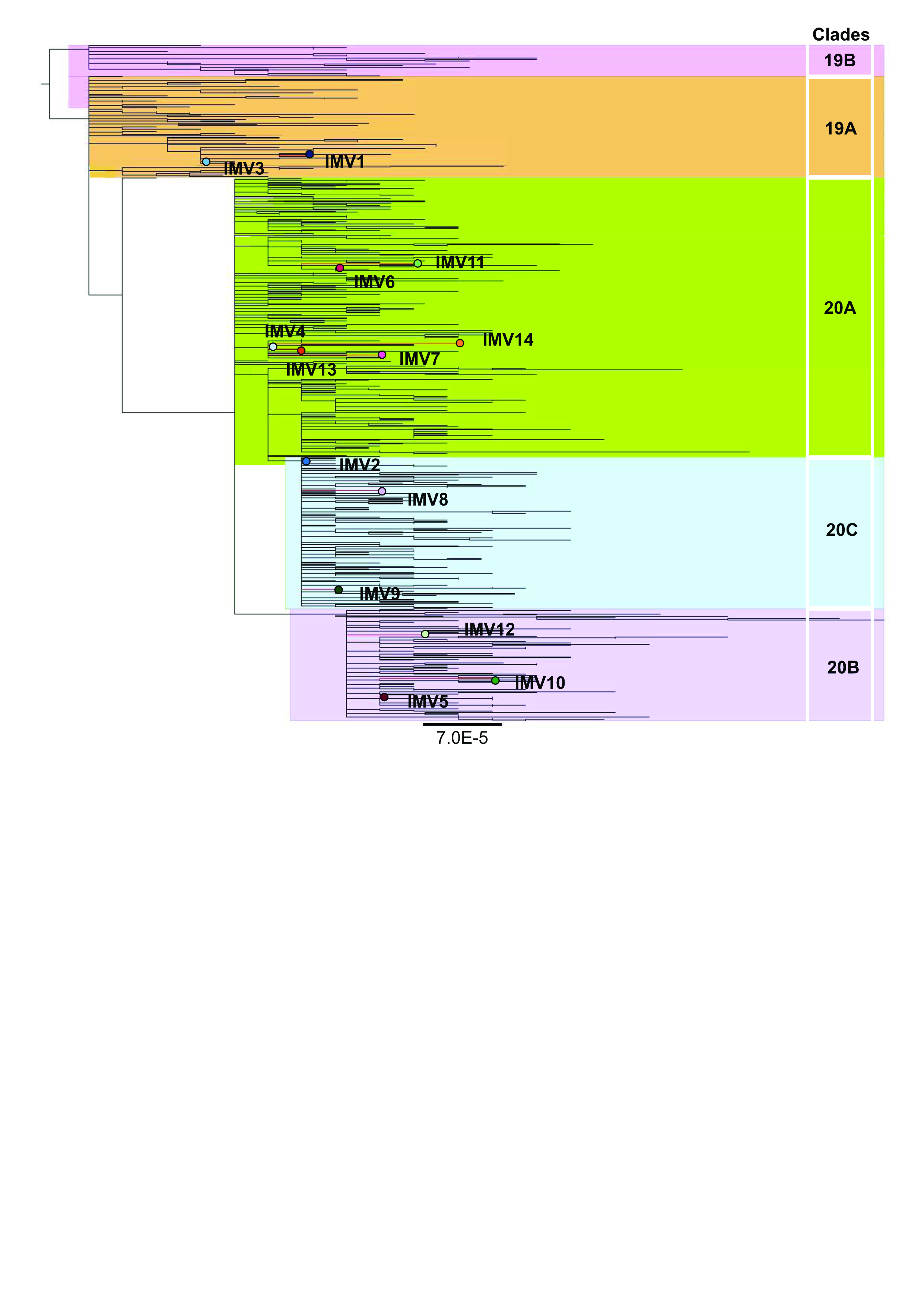

Supplement: S1 Fig — Phylogenetic analysis of sequences derived from the 14 SARS-CoV-2 P2 isolates, together with the SARS-CoV-2 sequences from Alm et al. [26] that represent viral diversity across the WHO European Region during the same time frame. Wuhan/WH04/2020 (EPI_ISL_406801), belonging to clade 19B, was chosen as the outgroup. The tree is colored based on SARS-CoV-2 Nextstrain clades, with circles indicating the positions of 14 SARS-CoV-2 isolates generated in this study. The scale bar indicates the number of nucleotide substitutions per site. SARS-CoV-2, Severe Acute Respiratory Syndrome Coronavirus 2. (TIF) [file pbio.3001006.s001.tif]

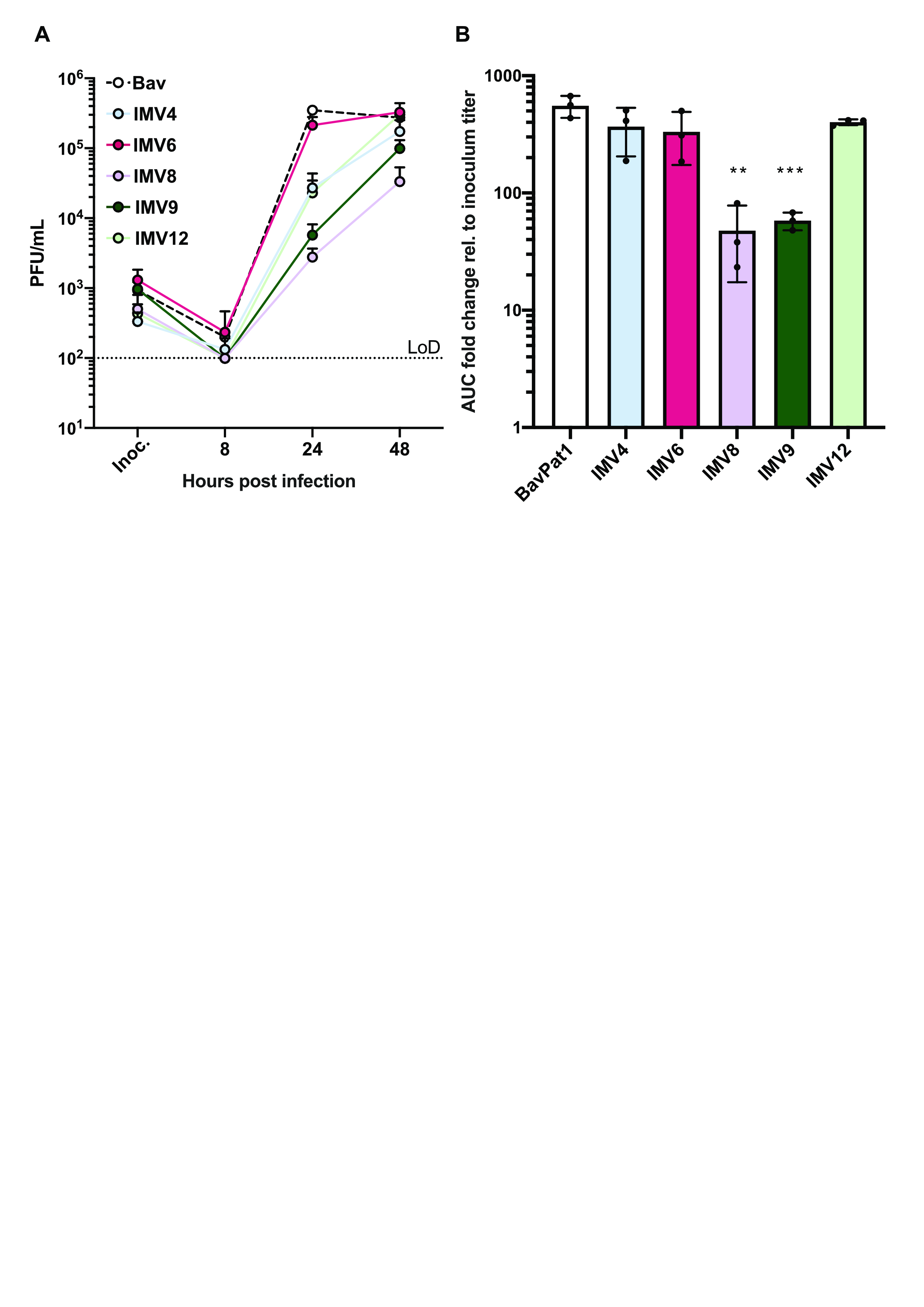

Supplement: S2 Fig — (A) Vero-E6 cells were infected with the indicated SARS-CoV-2 isolates at an MOI of 0.01 PFU/cell. Supernatants were harvested at the indicated times postinfection, and SARS-CoV-2 titers were determined by plaque assay. Data represent means and standard deviations from 3 independent experiments, with each experiment performed using 1 individual well. The dotted line crossing the y-axis at 102 PFU/mL indicates the assay LoD. (B) AUC values for the virus replication data shown in (A), normalized to inoculum titer of the respective isolate. Data represent means and standard deviations from the 3 independent experiments. A 1-way ANOVA was performed on log2-transformed data to test for significant differences between the individual isolates (p < 0.0001). To test for statistical significance against BavPat1, an unpaired t test was used on log2-transformed data (**p < 0.005; ***p < 0.0005). For underlying data, see S1 Data. AUC, area under the curve; LoD, limit of detection; MOI, multiplicity of infection; PFU, plaque-forming unit; SARS-CoV-2, Severe Acute Respiratory Syndrome Coronavirus 2. (TIF) [file pbio.3001006.s002.tif]

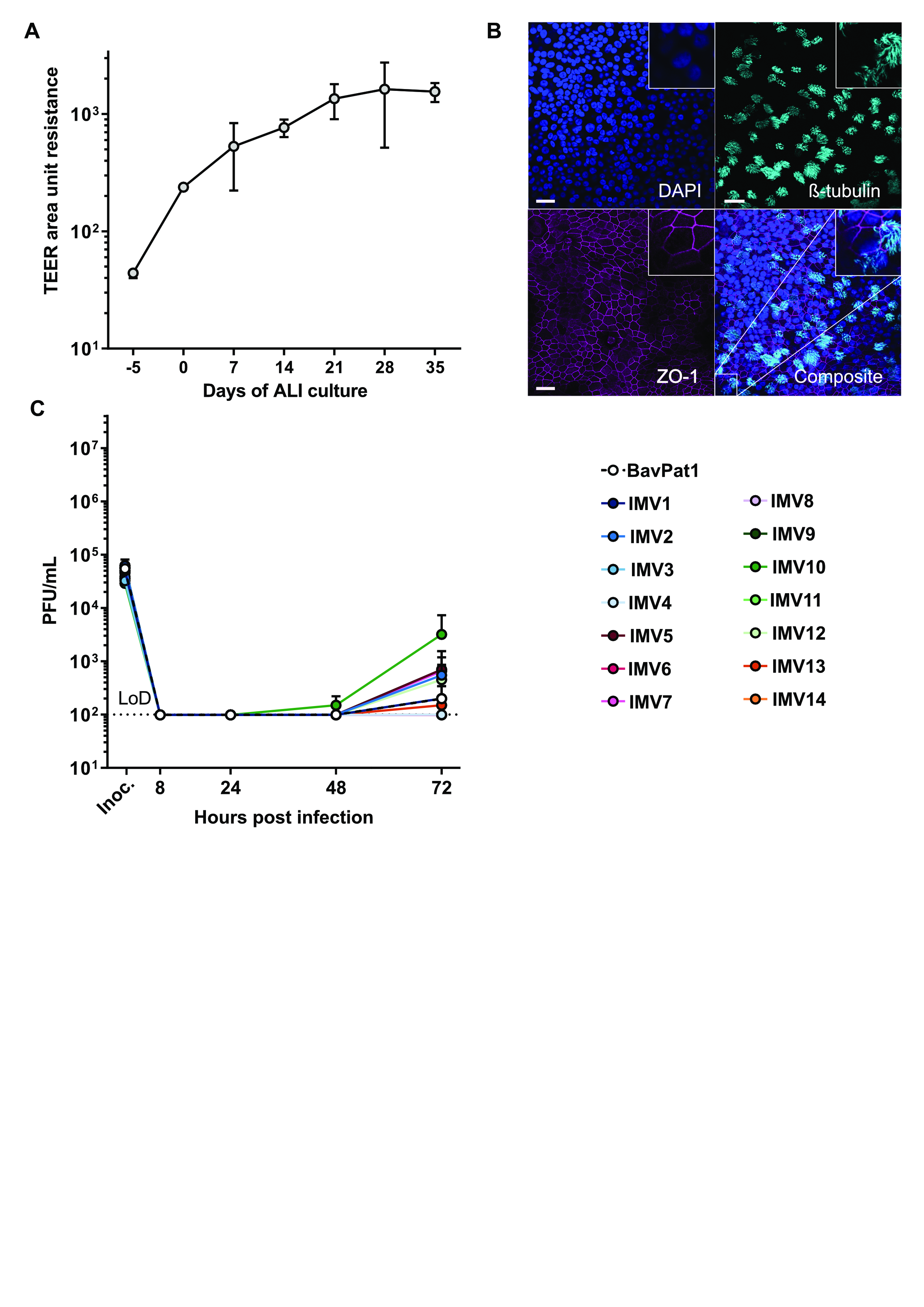

Supplement: S3 Fig — (A) During primary human BEpC (donor 1) differentiation into a pseudostratified airway epithelium at the ALI, TEER was measured weekly. Data represent mean TEER values and standard deviations from 3 independent wells at each time point. (B) Differentiated BEpCs from donor 1 were fixed and stained for ciliated cells (β-tubulin; cyan), tight junctions (ZO-1; magenta), and nuclei (DAPI; blue). Scale bar represents 25 μm. (C) Differentiated BEpCs from donor 1 were infected from the apical side with 6,000 PFU of each SARS-CoV-2 isolate (see Fig 2B). At the indicated times postinfection, basolateral samples were harvested, and virus titers were determined by plaque assay. Data represent means and standard deviations from 2 independent replicates. The dotted line crossing the y-axis at 102 PFU/mL indicates the assay LoD. For underlying data, see S1 Data. ALI, air–liquid interface; BEpC, bronchial epithelial cell; LoD, limit of detection; PFU, plaque-forming unit; SARS-CoV-2, Severe Acute Respiratory Syndrome Coronavirus 2; TEER, transepithelial electrical resistance; ZO-1, zona occludens protein 1. (TIF) [file pbio.3001006.s003.tif]

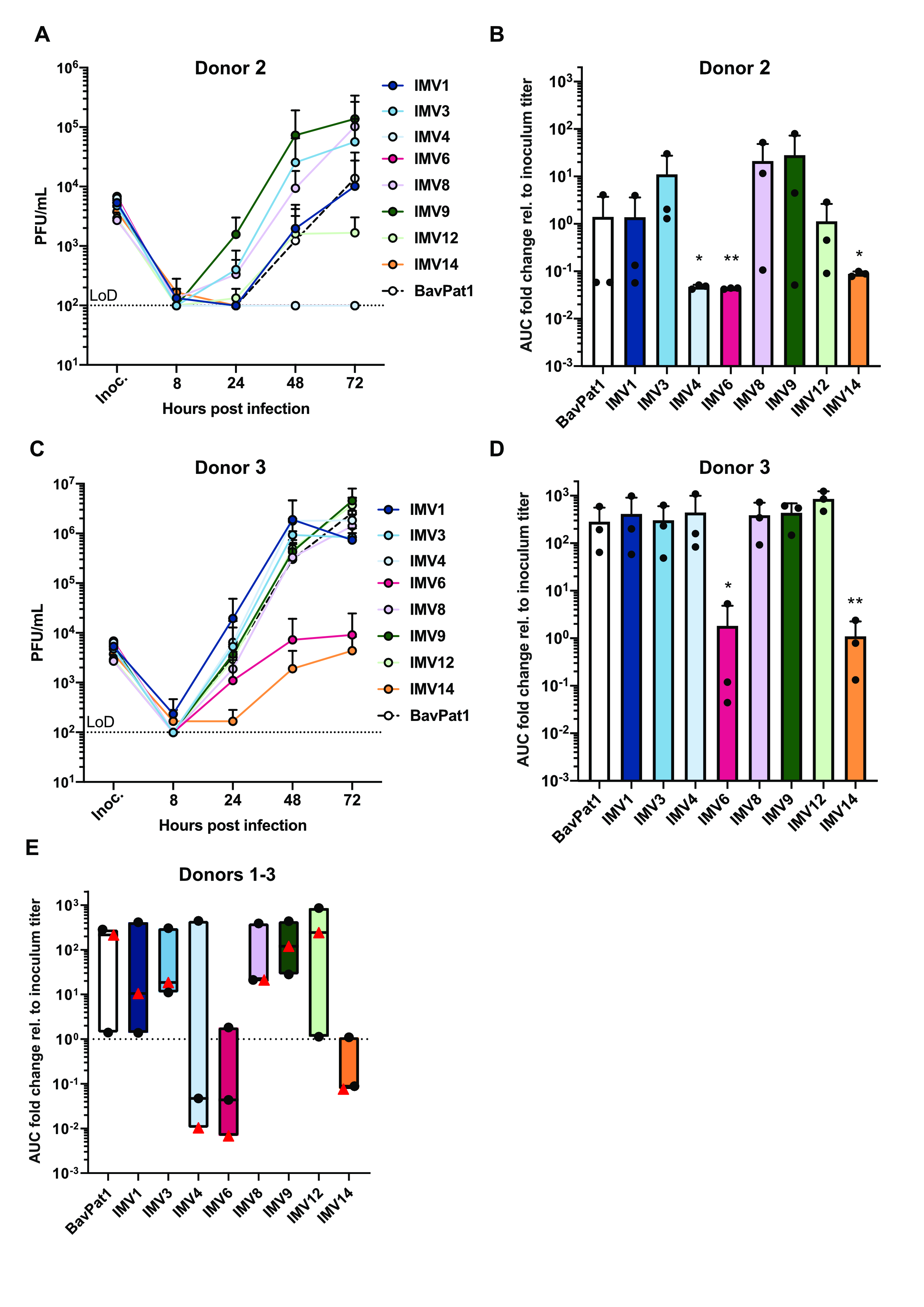

Supplement: S4 Fig — (A and C) Differentiated BEpCs from donors 2 (A) and 3 (C) were infected with 6,000 PFU of the indicated SARS-CoV-2 isolate from the apical side. At the indicated times postinfection, apical washes were harvested, and virus titers were determined by plaque assay. Data represent means and standard deviations from 3 independent replicates. The dotted lines crossing the y-axes at 102 PFU/mL indicate the assay LoD. (B and D) AUC values for the virus replication data shown in (A and C), normalized to inoculum titer of the respective isolate. Data represent means and standard deviations from the 3 independent experiments. A 1-way ANOVA was performed on log2-transformed data to test for significant differences between the individual isolates (n.s. for donor 2; p < 0.0001 for donor 3). To test for statistical significance against BavPat1 (D), an unpaired t test was used on log2-transformed data (*p < 0.05; **p < 0.01). Of note, as BavPat1 exhibited an unexplained attenuated phenotype in BEpCs from donor 2 (B), an unpaired t test was used on log2-transformed data to test for statistical significance against IMV3 (*p < 0.05; **p < 0.01). (E) Boxplot representation of the AUC values for the virus replication data from the 3 independent donors (data from B and D; and Fig 2C), normalized to inoculum titer of the respective isolate and donor. Shown is median and standard deviation. The red triangles represent data obtained from donor 1. For underlying data, see S1 Data. AUC, area under the curve; BEpC, bronchial epithelial cell; LoD, limit of detection; PFU, plaque-forming unit; SARS-CoV-2, Severe Acute Respiratory Syndrome Coronavirus 2. (TIF) [file pbio.3001006.s004.tif]

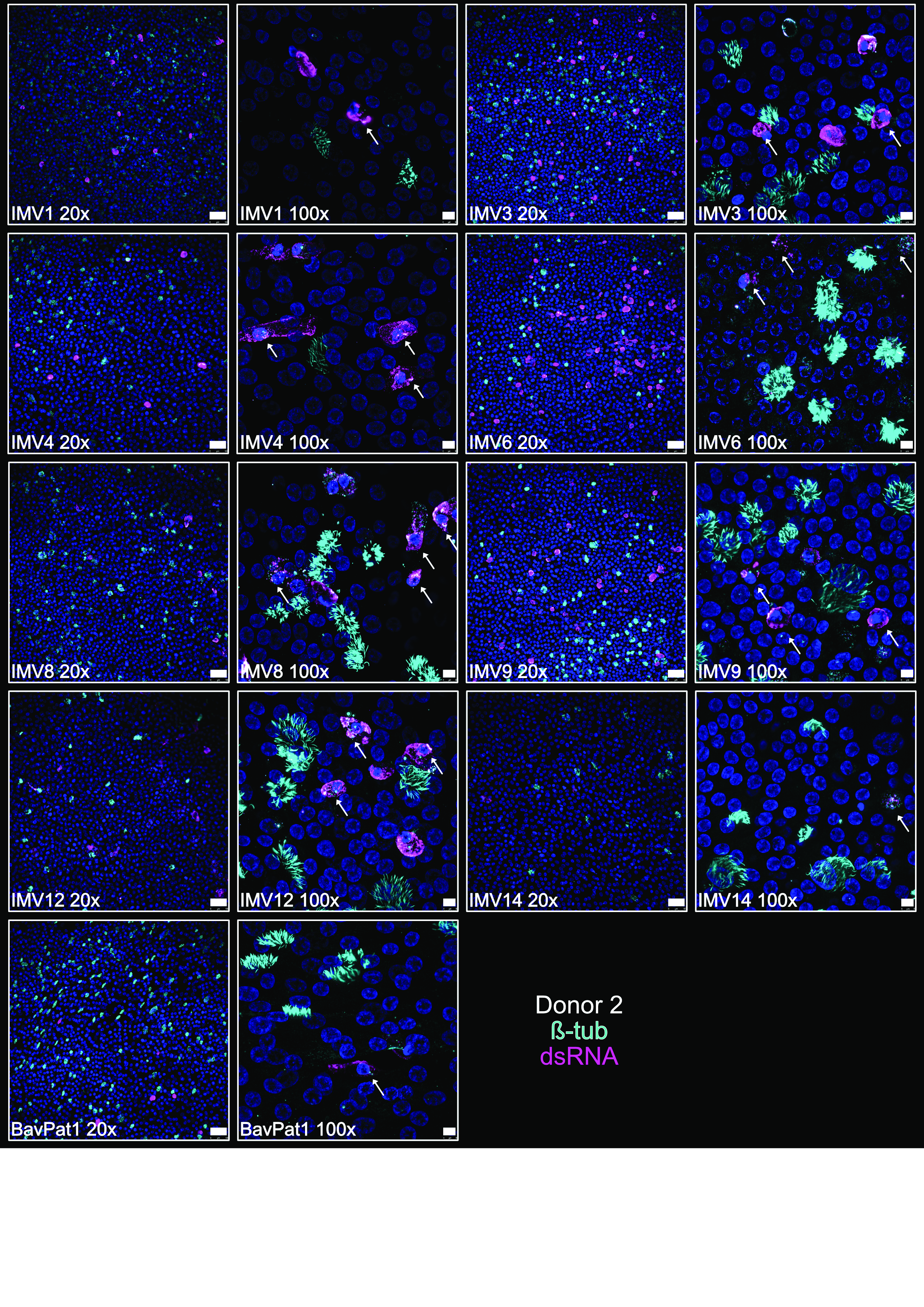

Supplement: S5 Fig — Differentiated BEpCs from donor 2 were infected from the apical side with 6,000 PFU of the indicated SARS-CoV-2 isolate. At 72 h postinfection, cells were fixed, permeabilized, and stained for the presence of infected cells (dsRNA; magenta) and ciliated cells (β-tubulin; cyan). Nuclei were stained with DAPI (blue). Arrows indicate co-localization. Scale bars represent 50 μm for the 20× magnifications and 8 μm for the 100× magnifications. Representative maximum projection images of z-stacks from one experiment are shown. BEpC, bronchial epithelial cell; PFU, plaque-forming unit; SARS-CoV-2, Severe Acute Respiratory Syndrome Coronavirus 2. (TIF) [file pbio.3001006.s005.tif]

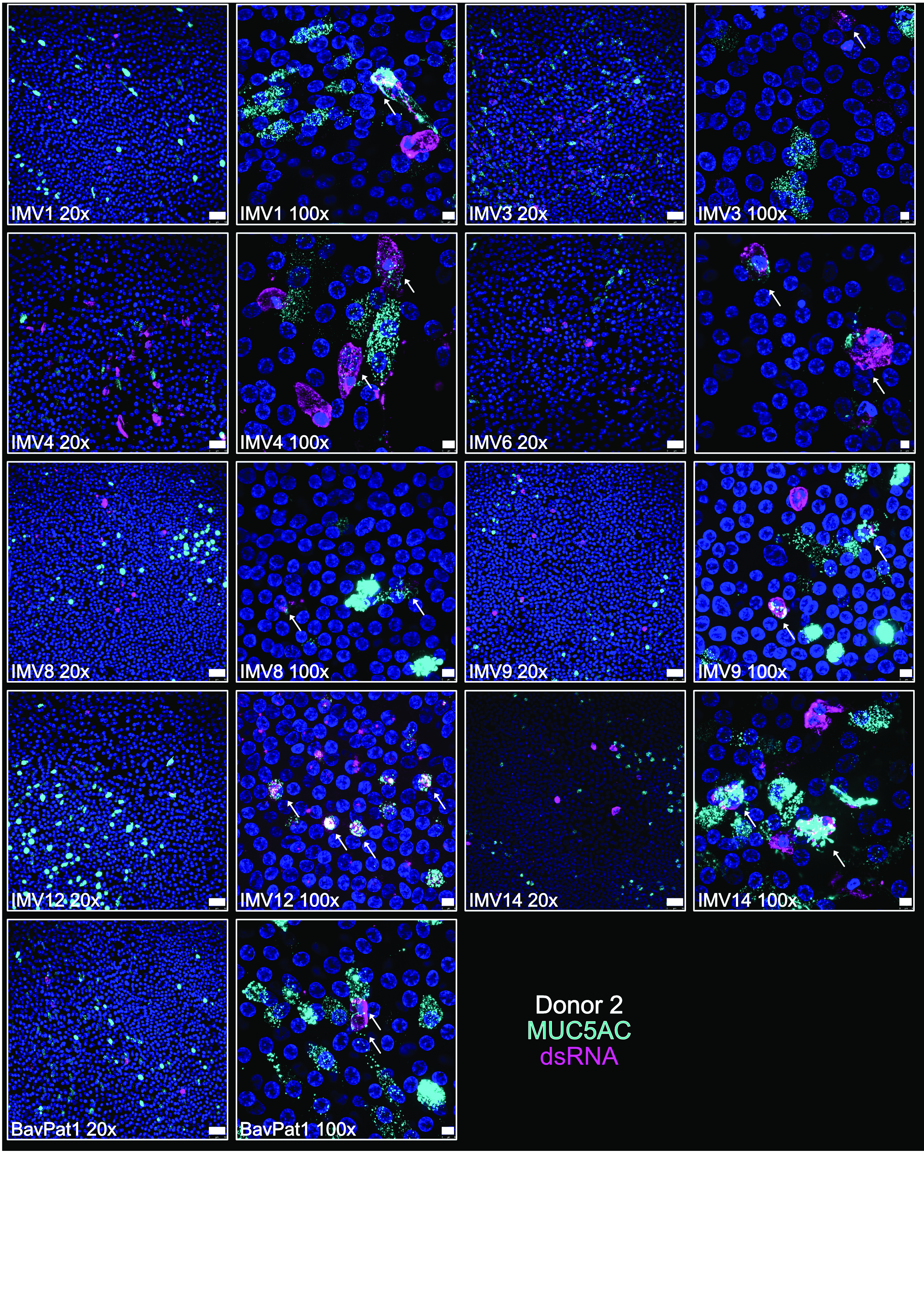

Supplement: S6 Fig — Differentiated BEpCs from donor 2 were infected from the apical side with 6,000 PFU of the indicated SARS-CoV-2 isolate. At 72 h postinfection, cells were fixed, permeabilized, and stained for the presence of infected cells (dsRNA; magenta) and goblet cells (MUC5AC; cyan). Nuclei were stained with DAPI (blue). Arrows indicate co-localization. Scale bars represent 50 μm for the 20× magnifications and 8 μm for the 100× magnifications. Representative maximum projection images of z-stacks from one experiment are shown. BEpC, bronchial epithelial cell; PFU, plaque-forming unit; SARS-CoV-2, Severe Acute Respiratory Syndrome Coronavirus 2. (TIF) [file pbio.3001006.s006.tif]

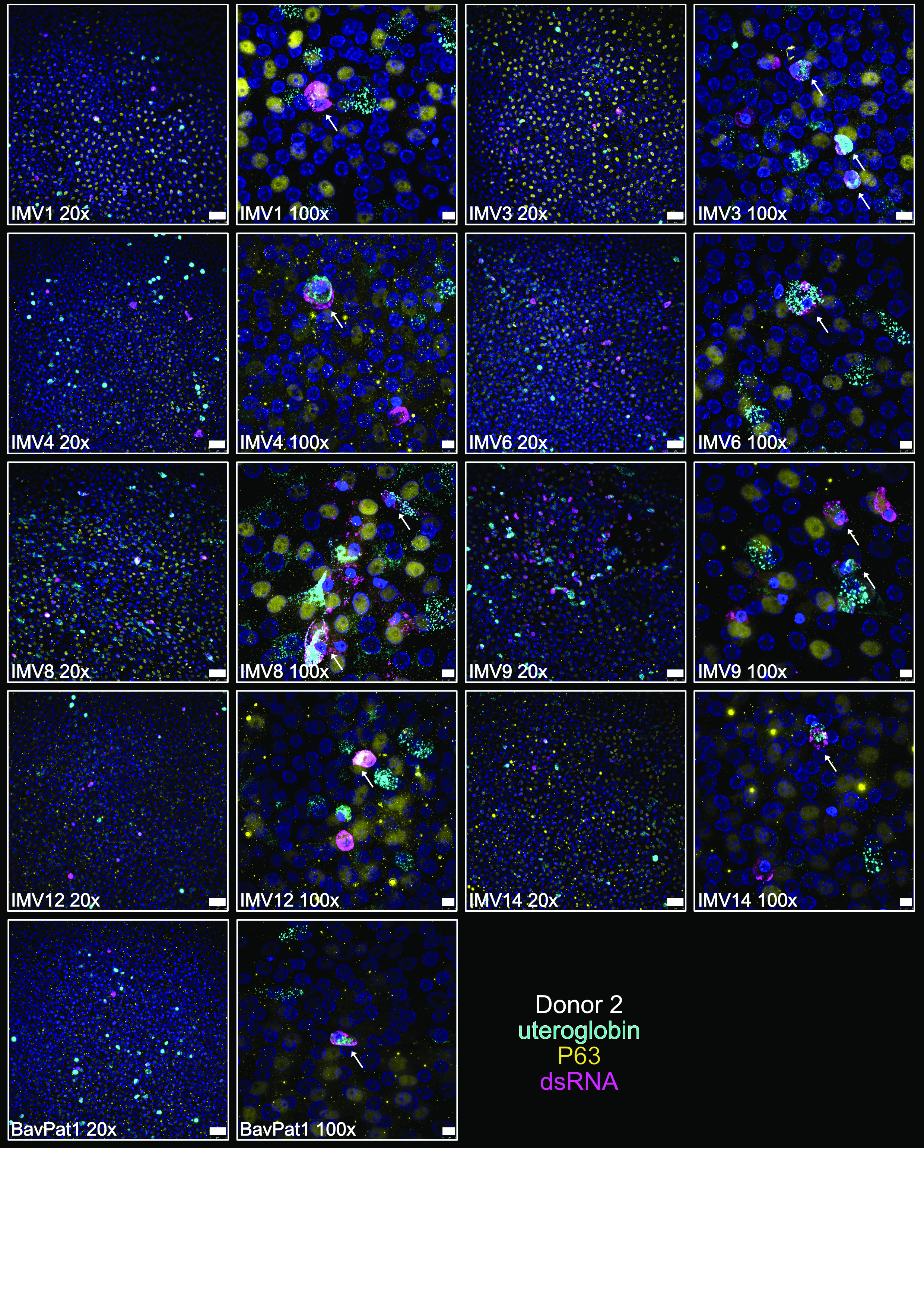

Supplement: S7 Fig — Differentiated BEpCs from donor 2 were infected from the apical side with 6,000 PFU of the indicated SARS-CoV-2 isolate. At 72 h postinfection, cells were fixed, permeabilized, and stained for the presence of infected cells (dsRNA; magenta), club cells (uteroglobin; cyan), and basal cells (P63; yellow). Nuclei were stained with DAPI (blue). Arrows indicate co-localization. Scale bars represent 50 μm for the 20× magnifications and 8 μm for the 100× magnifications. Representative maximum projection images of z-stacks from one experiment are shown. BEpC, bronchial epithelial cell; PFU, plaque-forming unit; SARS-CoV-2, Severe Acute Respiratory Syndrome Coronavirus 2. (TIF) [file pbio.3001006.s007.tif]

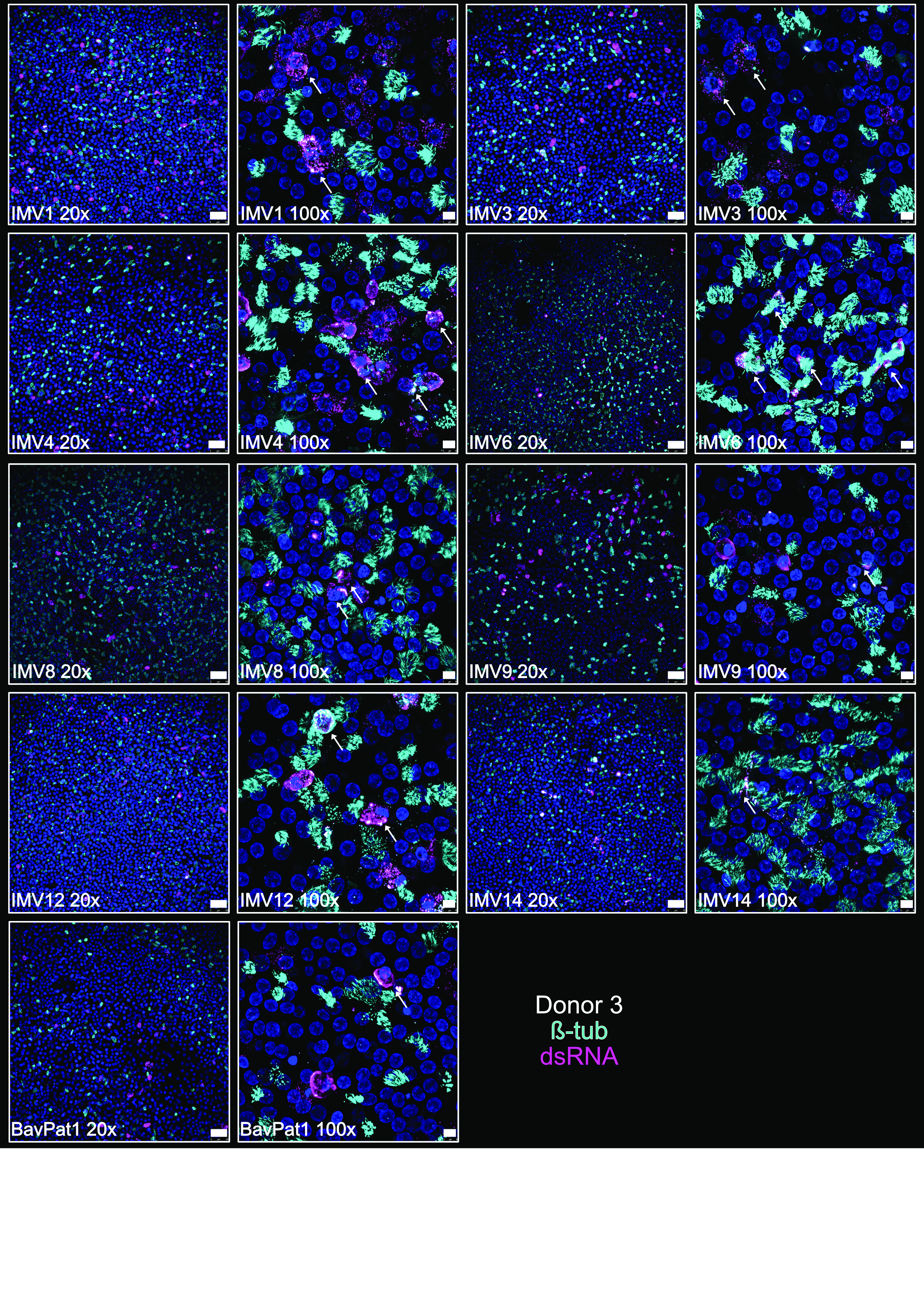

Supplement: S8 Fig — Differentiated BEpCs from donor 3 were infected from the apical side with 6,000 PFU of the indicated SARS-CoV-2 isolate. At 72 h postinfection, cells were fixed, permeabilized, and stained for the presence of infected cells (dsRNA; magenta) and ciliated cells (β-tubulin; cyan). Nuclei were stained with DAPI (blue). Arrows indicate co-localization. Scale bars represent 50 μm for the 20× magnifications and 8 μm for the 100× magnifications. Representative maximum projection images of z-stacks from one experiment are shown. BEpC, bronchial epithelial cell; PFU, plaque-forming unit; SARS-CoV-2, Severe Acute Respiratory Syndrome Coronavirus 2. (TIF) [file pbio.3001006.s008.tif]

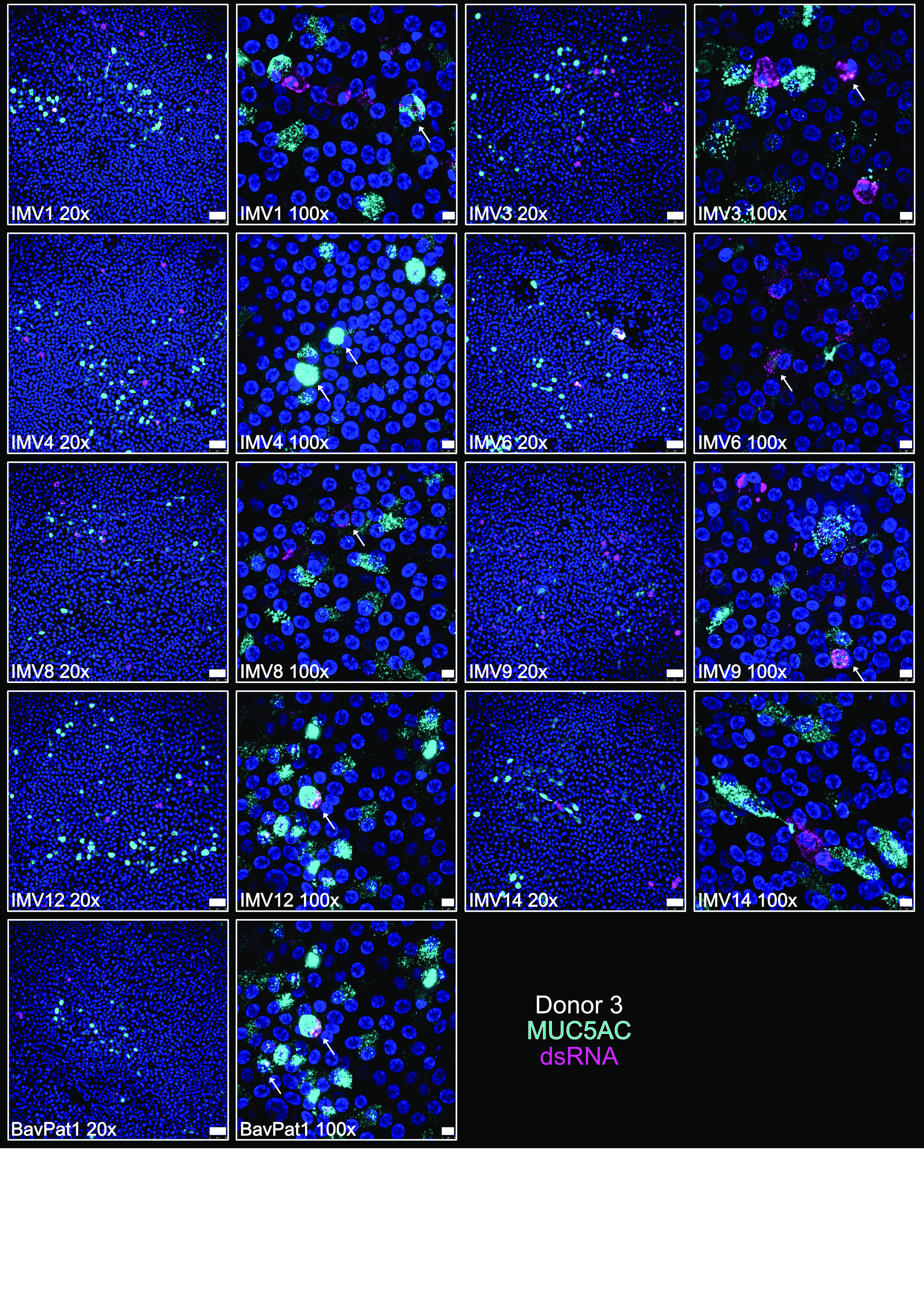

Supplement: S9 Fig — Differentiated BEpCs from donor 3 were infected from the apical side with 6,000 PFU of the indicated SARS-CoV-2 isolate. At 72 h postinfection, cells were fixed, permeabilized, and stained for the presence of infected cells (dsRNA; magenta) and goblet cells (MUC5AC; cyan). Nuclei were stained with DAPI (blue). Arrows indicate co-localization. Scale bars represent 50 μm for the 20× magnifications and 8 μm for the 100× magnifications. Representative maximum projection images of z-stacks from one experiment are shown. BEpC, bronchial epithelial cell; PFU, plaque-forming unit; SARS-CoV-2, Severe Acute Respiratory Syndrome Coronavirus 2. (TIF) [file pbio.3001006.s009.tif]

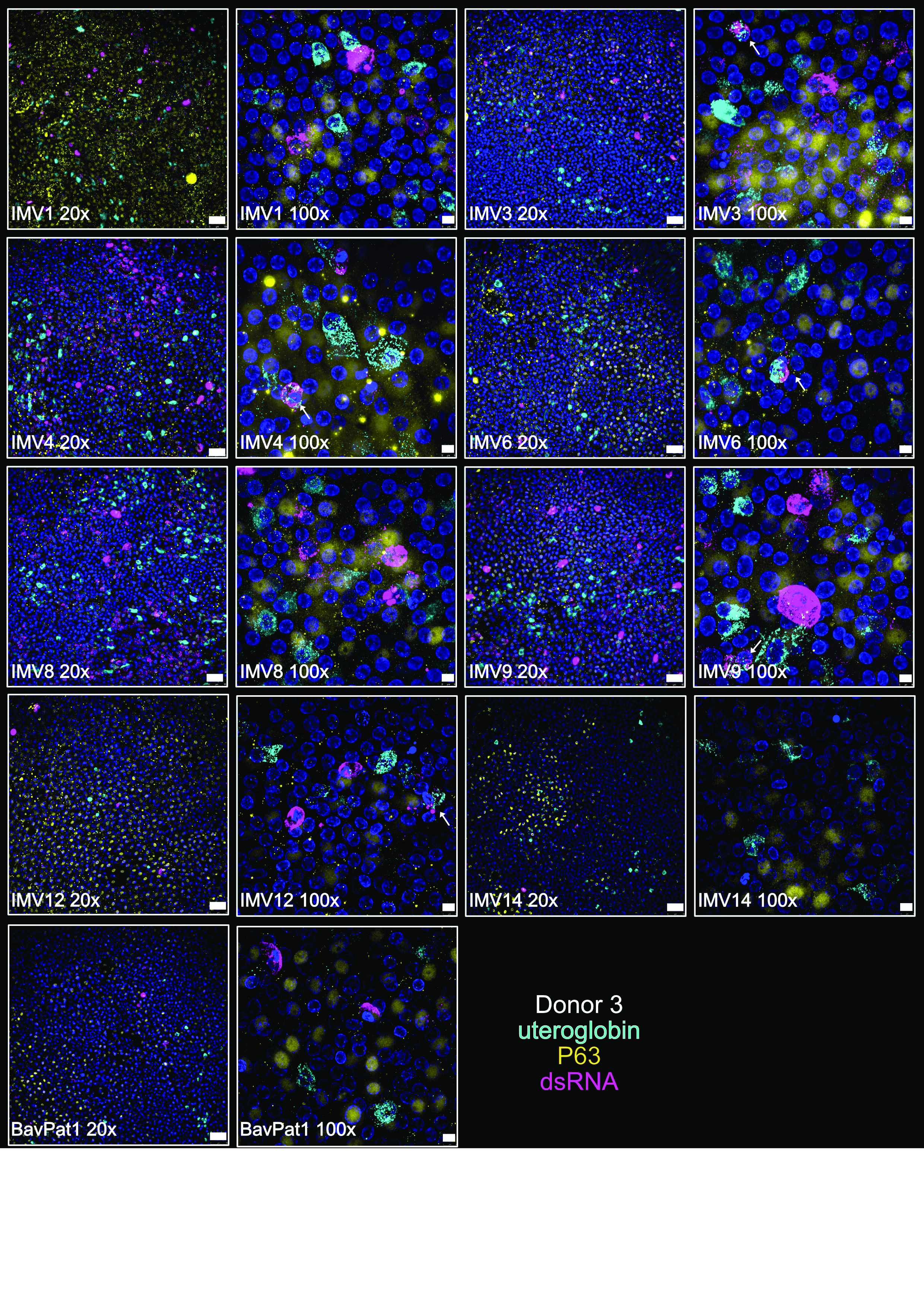

Supplement: S10 Fig — Differentiated BEpCs from donor 3 were infected from the apical side with 6,000 PFU of the indicated SARS-CoV-2 isolate. At 72 h postinfection, cells were fixed, permeabilized, and stained for the presence of infected cells (dsRNA; magenta), club cells (uteroglobin; cyan), and basal cells (P63; yellow). Nuclei were stained with DAPI (blue). Arrows indicate co-localization. Scale bars represent 50 μm for the 20× magnifications and 8 μm for the 100× magnifications. Representative maximum projection images of z-stacks from one experiment are shown. BEpC, bronchial epithelial cell; PFU, plaque-forming unit; SARS-CoV-2, Severe Acute Respiratory Syndrome Coronavirus 2. (TIF) [file pbio.3001006.s010.tif]
