# Supplementary material for: SARS-CoV-2 variants reveal features critical for replication in primary human cells
Source: PLoS Biol. 2021 Mar 24;19(3):e3001006. doi: 10.1371/journal.pbio.3001006 (PMC8021179; doi:10.1371/journal.pbio.3001006)
Supplement: S1 Table — Ct, cycle threshold; SARS-CoV-2, Severe Acute Respiratory Syndrome Coronavirus 2. (PDF) [file pbio.3001006.s011.pdf]

Sample number	Ct custom PCR (E)	Ct Cobas (ORF1/E)	Success/ Failure	Sample number	Ct custom PCR (E)	Ct Cobas / ORF1/E)	Success/ Failure
1	17.86	-	success	34	-	30.91/33.24	failure
2	19.4	-	success	35	-	32.35/33.52	failure
3	18.95	-	failure	36	-	32.58/34.62	failure
4	19.98	-	success	37	-	32.34/33.37	failure
5	18.56	-	failure	38	-	30.97/31.44	failure
6	19.5	-	failure	39	-	32.21/33.62	failure
7	19.73	-	failure	40	-	33.33/34.8	failure
8	19.34	-	failure	41	-	30.88/32.18	failure
9	19.4	-	success	42	-	31.82/33.3	failure
10	17	-	success	43	-	neg/37.85	failure
11	18.5	-	success	44	-	34.93/36.44	failure
12	-	19.92/19.98	failure	45	37.42	-	failure
13	19.4	-	success	46	-	35.09/neg	failure
14	-	17.97/18.22	success	47	-	33.79/37.08	failure
15	21.75	-	success	48	-	35.23/38.12	failure
16	21	-	success	49	-	neg/38.71	failure
17	-	21.67/21.46	success	50	-	33.67/35.09	failure
18	-	21.35/21.5	failure	51	-	33.66/35.86	failure
19	-	20.04/20.16	success	52	-	35.12/36.64	failure
20	-	20.05/20.22	success	53	-	34.31/37.74	failure
21	-	24.81/24.47	failure	54	-	32.6/35.16	failure
22	-	28.73/29	failure	55	-	34.5/36.28	failure
23	-	28.96/29.48	success	56	-	34.44/37.54	failure
24	-	28.58/29.16	failure	57	-	34.12/36.14	failure
25	-	28.48/29.23	failure	58	-	neg/36.95	failure
26	-	29.67/29.91	failure	59	-	neg/37.96	failure
27	-	26.55/26.82	failure	60	-	neg/37.25	failure
28	-	28.8/28.92	failure	61	-	34.71/35.97	failure
29	-	27.7/27.96	failure	62	-	neg/36.68	failure
30	-	27.39/27.91	failure	63	-	33.79/37.08	failure
31	-	25.39/25.64	failure	64	-	33.67/35.09	failure
32	-	31.97/34.11	failure	65	-	33.66/35.86	failure
33	-	30.27/31.49	failure	66	-	32.6/35.16	failure
				67	-	33.28/35.2	failure

S1 Table. Cycle threshold (Ct) values, and success/failure outcomes of virus isolation attempts, from 67 patient samples that were PCR-positive for SARS-CoV-2
